# Mechanisms Mediating Spinal and Bulbar Muscular Atrophy: Investigations into Polyglutamine-Expanded Androgen Receptor Function and Dysfunction

**DOI:** 10.3389/fneur.2013.00053

**Published:** 2013-05-15

**Authors:** Lenore K. Beitel, Carlos Alvarado, Shaza Mokhtar, Miltiadis Paliouras, Mark Trifiro

**Affiliations:** ^1^Lady Davis Institute for Medical Research, Jewish General HospitalMontreal, QC, Canada; ^2^Department of Medicine, McGill UniversityMontreal, QC, Canada; ^3^Department of Human Genetics, McGill UniversityMontreal, QC, Canada

**Keywords:** androgen receptor, spinal and bulbar muscular atrophy, Kennedy’s disease, polyglutamine disease, neuromuscular disorder, mouse models, loss-of-function, gain-of-function

## Abstract

Spinal and bulbar muscular atrophy (SBMA, Kennedy’s disease), a late-onset neuromuscular disorder, is caused by expansion of the polymorphic polyglutamine tract in the androgen receptor (AR). The AR is a ligand-activated transcription factor, but plays roles in other cellular pathways. In SBMA, selective motor neuron degeneration occurs in the brainstem and spinal cord, thus the causes of neuronal dysfunction have been studied. However, pathogenic pathways in muscles may also be involved. Cultured cells, fly and mouse models are used to study the molecular mechanisms leading to SBMA. Both the structure of the polyglutamine-expanded AR (polyQ AR) and its interactions with other proteins are altered relative to the normal AR. The ligand-dependent translocation of the polyQ AR to the nucleus appears to be critical, as are interdomain interactions. The polyQ AR, or fragments thereof, can form nuclear inclusions, but their pathogenic or protective nature is unclear. Other data suggests soluble polyQ AR oligomers can be harmful. Post-translational modifications such as phosphorylation, acetylation, and ubiquitination influence AR function and modulate the deleterious effects of the polyQ AR. Transcriptional dysregulation is highly likely to be a factor in SBMA; deregulation of non-genomic AR signaling may also be involved. Studies on polyQ AR-protein degradation suggest inhibition of the ubiquitin proteasome system and changes to autophagic pathways may be relevant. Mitochondrial function and axonal transport may also be affected by the polyQ AR. Androgens, acting through the AR, can be neurotrophic and are important in muscle development; hence both loss of normal AR functions and gain of novel harmful functions by the polyQ AR can contribute to neurodegeneration and muscular atrophy. Thus investigations into polyQ AR function have shown that multiple complex mechanisms lead to the initiation and progression of SBMA.

## Introduction

Genetic alterations in the X-linked androgen receptor (AR) gene are associated with spinal and bulbar muscular atrophy (SBMA, Kennedy’s disease), an adult-onset neuromuscular disease, androgen insensitivity syndrome (AIS), and prostate cancer (Gottlieb et al., 2004, 2012). Although *AR* point mutations, insertions, and deletions lead to varying degrees of AIS, the underlying cause of SBMA is an expansion of the polymorphic *AR* CAG repeat encoding a glutamine tract (La Spada et al., 1991). As a result, the AR protein contains an expanded polyglutamine tract (*n* ≥ 38) that alters the AR’s normal functions and physiological roles. This article refers to the polyglutamine-expanded AR as the polyQ AR, in order to differentiate it from mutant ARs found in other diseases, and primarily focuses on investigations into the molecular pathogenesis of SBMA published since our last review (Beitel et al., 2005).

SBMA is one of at least nine neurodegenerative diseases caused by expansion of a polyglutamine tract within a specific protein. Others include Huntington’s disease (HD), dentatorubral-pallidoluysian atrophy (DRPLA), and six spinocerebellar ataxias [SCA1, SCA2, SCA3 (Machado-Joseph disease), SCA-6, SCA-7, SCA-17 (Truant et al., 2006; Orr and Zoghbi, 2007; La Spada and Taylor, 2010)]. Except for SBMA, which is X-linked, these disorders are inherited in an autosomal-dominant fashion. SBMA has a prevalence of about 1 in 50,000 males, HD, 1 per 15,000 persons worldwide, and, all together, the prevalence of the SCAs has been estimated at ∼3 per 100,000 (Guyenet and La Spada, 2006; Truant et al., 2006). The polyglutamine diseases share several features including late-onset, progressive neurodegeneration, accumulation of misfolded polyQ proteins in the cytoplasm or nucleus of neurons, and a positive correlation between CAG repeat length and disease severity (Parodi and Pennuto, 2011; Tanaka et al., 2012). Despite widespread expression of the polyQ proteins, only specific neuronal populations are affected in each disease. Even so, common mechanisms that disturb neuronal functions in the polyQ disorders may be identified, leading to the development of effective therapies for these diseases (Pennuto and Fischbeck, 2010; Takahashi et al., 2010).

## Androgen Receptor Structure and Mechanisms of Action

**Figure 1:**
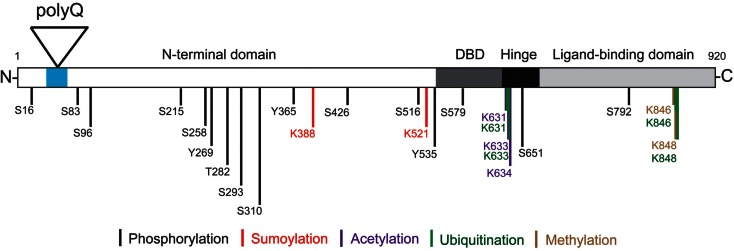
**Androgen receptor structure and post-translational modifications**. The locations of the AR N-terminal domain (NTD), polyglutamine tract (polyQ), DNA-binding domain (DBD), hinge region, and C-terminal ligand-binding domain (LBD) are shown. Phosphorylation can occur on serine (S), threonine (T), and tyrosine (Y) residues. Specific lysine (K) residues can be acetylated, ubiquitinated, sumoylated, or methylated as indicated. Note the amino acid numbering is based on a 920 amino acid AR (NCBI Reference Sequence: NM_000044.3) (Gottlieb et al., 2012).

The AR is a member of the nuclear receptor superfamily and has been well characterized as a ligand-activated transcription factor (Brinkmann, 2011). It contains an N-terminal domain (NTD) that modulates transcriptional activation, a central DNA-binding domain (DBD) that binds androgen-response elements (AREs), and a C-terminal ligand-binding domain (LBD) (Figure 1). Subdomains involved in nuclear localization, dimerization, and interaction with heat-shock proteins (HSPs), co-activators, and other proteins have also been identified (Centenera et al., 2008; Parodi and Pennuto, 2011). Typically, the AR resides in the cytoplasm of the cell in the absence of androgens, complexed with HSP (Brinkmann, 2011). Once the receptor binds testosterone, or its metabolite, 5α-dihydrotestosterone (DHT), the AR undergoes a conformational change that promotes heat-shock protein dissociation, and exposes its nuclear localization signal (NLS), DNA-binding, and dimerization domains. Following nuclear translocation, ARs dimerize at AREs, recruit coregulators, and transactivate AR target genes. The resulting ligand-dependent changes in mRNA and protein expression are thought to be responsible for the differential physiological actions of testosterone and DHT (Brinkmann, 2011). However, a proteomics/systems biology approach to identifying proteins within AR complexes found that the wild-type AR (wt AR) interacts with a wide variety of proteins involved in RNA splicing, protein translation, proteasome/protein ubiquitination, and transcription, suggesting the AR participates in numerous cellular pathways (Paliouras et al., 2011).

## Androgen Insensitivity Syndrome and SBMA

The AR plays a role in male sexual differentiation, and the development and function of nerves, brain, testes, prostate, and muscles (Jordan and Doncarlos, 2008; MacLean et al., 2008). In XY individuals, the spectrum of AIS ranges from complete androgen insensitivity, which results in a female phenotype, to mild resistance to androgens, which leads to undervirilization, breast enlargement (gynecomastia), and/or infertility in males (Brinkmann, 2001). Conversely, men with SBMA generally develop an adult-onset disorder with progressive muscle cramps, muscle weakness and wasting, muscle twitching (fasciculations), dysarthria (difficulty articulating), and dysphagia (difficulty swallowing) (Rhodes et al., 2009; Katsuno et al., 2010b), in addition to signs of mild AIS. In some cases, these patients are initially misdiagnosed with amyotrophic lateral sclerosis (ALS; Lou Gehrig’s disease), however, genetic testing for an expanded *AR* CAG repeat (*n* ≥ 38) confirms a diagnosis of SBMA (Parboosingh et al., 1997; Bruson et al., 2012). This differential diagnosis is critical, as ∼50% of ALS patients die within 3 years of disease onset, with involvement of both upper and lower motor neurons (Mitchell and Borasio, 2007), whereas the life expectancy for SBMA patients is normal or only minimally reduced (Chahin et al., 2008).

Although men with SBMA may exhibit certain symptoms of AIS [e.g., gynecomastia and reduced fertility (Dejager et al., 2002)], which have been attributed to AR loss-of-function, individuals with AIS do not display the neuromuscular phenotype associated with SBMA. Therefore, the polyQ AR must also gain novel functions that are selectively harmful to motor neurons (Trifiro et al., 1994), and, as shown recently, detrimental to skeletal muscles. SBMA is characterized by loss of lower motor neurons from the brain stem and anterior horn of the spinal cord, which has been presumed to lead to progressive muscle wasting (Ross, 1995). Accordingly, extensive research has focused on mechanisms by which the polyQ AR causes neuronal dysfunction and degeneration (Walcott and Merry, 2002; Beitel et al., 2005; Cary and La Spada, 2008; Finsterer, 2009), and, more recently, pathogenic pathways in muscles (Yu et al., 2006a; Monks et al., 2007; Palazzolo et al., 2009). SBMA is unique among the polyglutamine diseases in that it is a ligand (androgen)-dependent disorder (Katsuno et al., 2002; Orr and Zoghbi, 2007). Thus, men expressing the polyQ AR exhibit progressive neuromuscular signs, while women who are heterozygous for the polyQ AR mutation develop very mild symptoms due to and lower levels of circulating AR ligands (testosterone and DHT) (Chevalier-Larsen and Merry, 2012). SBMA has been found in men from various ethnic backgrounds throughout the world, although the prevalence is higher in certain regions of Japan and Western Finland (Finsterer, 2010; Katsuno et al., 2010b).

## Model Systems

A number of systems have been used to investigate the molecular and cellular mechanisms underlying SBMA, ranging from cultured cells to fly and mouse models [summarized in Table 1; mouse models reviewed in (Merry, 2005; Jordan and Lieberman, 2008; Figiel et al., 2012)]. Cells that lack AR are transiently or stably transfected to examine the effects of the wt or polyQ AR. PC12 cells that inducibly express wt and polyQ ARs have been used extensively (e.g., Montie and Merry, 2009; Ranganathan et al., 2009; Orr et al., 2010; Montie et al., 2011). In *Drosophila*, ARs can be expressed in a tissue-specific fashion, depending on the promoter used to drive AR expression. Most SBMA mouse models, however, are based on overexpression of a polyQ AR protein with more than 90Q; these mice also express endogenous wt AR (Katsuno et al., 2002; McManamny et al., 2002; Chevalier-Larsen et al., 2004; Sopher et al., 2004). The transgenic AR97Q mice express the full-length human AR (hAR) containing 97 CAG repeats under the control of a cytomegalovirus enhancer and a chicken β-actin promoter (Katsuno et al., 2002), while the prion protein promoter was used to drive expression of the hAR with 112Q in the AR112Q transgenic mice (Chevalier-Larsen et al., 2004). In contrast, a yeast artificial chromosome (YAC) genomic fragment containing the 100 kb hAR gene with 100 CAG repeats was used to generate the transgenic AR100 model mice (Sopher et al., 2004) (Table 1). SBMA patients, however, express only a polyQ AR protein with 38–62 Gln, and lack wt AR. The presence of the wt AR in transgenic mice models may moderate or compensate for the effects of the polyQ AR. This possibility was highlighted when the transgenic AR100 YAC mice were crossed with AR null testicular feminization mice (*Tfm)* (Table 1) (Thomas et al., 2006b), as the AR100Tfm mice exhibited an accelerated neuromuscular disease phenotype relative to the AR100 mice. As well, although the AR20 protein in AR20Tfm mice was able to partially rescue the female-like Tfm phenotype, the external genitalia of the AR100Tfm mice was the same as female or androgen-insensitive Tfm mice, consistent with a loss-of-function of the polyQ AR protein.

**Table 1:** **Cell and animal models of SBMA**.

Cell line	Description		Features			Reference
HEK293	Human embryonic kidney cells					
HeLa	Human cervical cancer cells					
MN AR24; MN AR65	Mouse hybrid motoneuron-neuroblastoma cells		MN-1 cells stably transfected with hAR 24Q or 65Q			Brooks et al. (1997)
NSC34	Mouse neuroblastoma-spinal cord hybrid cell line		Resemble developing motor neurons.			Cashman et al. (1992)
PC12	Rat adrenal pheochromocytoma-derived cell line		Model system for neuronal differentiation			Greene and Tischler (1976)
SK-N-SH; SH-SY5Y	Human neuroblastoma cell lines		General neuron-like phenotype			Ross et al. (1983)

***Drosophila***	**Description**		**Features**			**Reference**

hAR(Q52)	UAS-hAR(Q52); GMR-GAL4		ARQ52 expressed in eye photoreceptors neuron under control of *GMR* promote*r*			Takeyama et al. (2002)
ARQ77	UAS-ARQ77; ELAV-GAL4		ARQ77 expressed throughout CNS under control of pan-neuronal *ELAV* promoter			Funderburk et al. (2009)
ARQ65	pUAST-ARQ65; OK371-GAL4		ARQ65 expressed in a defined subset of neurons under control of OK371 promoter			Jochum et al. (2012)

**Mouse model**	**AR/promoter**	**Neuronal pathology**	**Nuclear inclusions**	**Muscle pathology**	**Other features**	**Reference**

Transgenic AR120Q	Full-length hAR (120Q); CMV promoter	Yes	No	Yes	Muscle weakness & atrophy; testicular atrophy	McManamny et al. (2002)
Transgenic AR97Q	Full-length hAR (97Q); chicken β-actin promoter, CMV enhancer	Yes	Yes, motor neurons, spinal cord, CNS, muscle, etc.	Yes	Muscle weakness & atrophy; neurogenic & myogenic myopathy	Katsuno et al. (2002)
Transgenic AR100	Complete hAR gene (100Q) in YAC	Yes	No, motor neurons; Yes, CNS, liver, muscle	Yes	Muscle weakness & hindlimb atrophy	Sopher et al. (2004)
Transgenic AR112Q	Full-length hAR (112Q); prion protein promoter	No	Yes, spinal cord, brain stem & cortex	No	Hindlimb muscle weakness; some male infertility	Chevalier-Larsen et al. (2004)
Knock-in AR113Q	1340 bp of mAR exon 1 replaced by hAR (113Q) exon 1	Yes	Yes, CNS, muscle	Yes	Motor deficits; testicular atrophy	Albertelli et al. (2006), Yu et al. (2006a)
Transgenic HSA-AR	Rat AR(22Q); HSA promoter	Axonopathy	No	Yes	Overexpress AR only in skeletal muscle	Monks et al. (2007)
Tfm	AR null	No	No	No	Model for AIS	Gaspar et al. (1991)

*CMV, cytomegalovirus; CNS, central nervous system; ELAV, embryonic lethal abnormal visual system promoter, hAR, human AR; HSA, human skeletal actin promoter; mAR, mouse AR; YAC, yeast artificial chromosome*.

A knock-in mouse model for SBMA was made by replacing 1,340 bp of the coding sequence of mouse AR exon 1 with hAR exon 1 sequence containing 113 CAGs (Albertelli et al., 2006; Yu et al., 2006a) (Table 1). In contrast to the AR100Tfm mice, in the transgenic AR113Q mice, the polyQ AR was able to effectively masculinize the male mice, likely because the AR113Q is under the regulatory control of the mouse AR gene promoter. The AR113Q knock-in mice show signs of partial androgen insensitivity, including testicular atrophy and decreased fertility, which are seen in SBMA patients (Yu et al., 2006b). A striking age-dependent testicular pathology was observed in the AR113Q, which was distinct from the testicular atrophy seen in *Tfm* mice. These results lead to the conclusion that the abnormalities in testicular morphology in AR113Q were mediated not only by a partial loss of AR function, but also reflect a “toxic” gain-of-function due to AR polyglutamine tract expansion. Unexpectedly, transgenic mice that overexpress the WT rat AR (22 Q) under control of the human skeletal α-actin (HSA) promoter solely in their skeletal muscle fibers reproduced many neuromuscular features of SBMA model mice (Monks et al., 2007). The neuronal and muscular pathology, the localization of nuclear inclusion and notable phenotype features of each mouse model are summarize in Table 1.

Testosterone plays a crucial role in progression of symptoms in SBMA mice models. However, raising testosterone levels in male AR112Q transgenic mice for 6 months did not worsen severity or age of onset of their disease, suggesting that the pathogenic mechanism of disease in SBMA saturates at close to endogenous testosterone levels (Chevalier-Larsen and Merry, 2012).

## Mechanisms Contributing to SBMA

Recent investigations have focused on a number of complex, and not necessarily independent, mechanisms that lead to the development of SBMA. These include alterations in AR structure, interaction of the polyQ AR with other proteins, transcriptional dysregulation, formation of harmful polyQ AR oligomers, changes in post-translational modifications, loss of neurotrophic support, and mitochondrial dysfunction. The possibility that polyQ AR expression in skeletal muscles contributes to SBMA is intriguing. More controversial are the role of nuclear inclusions, altered axonal transport, and inhibition of the ubiquitin proteasome system. These topics will be discussed in greater detail below.

## Alterations in AR Structure

Investigating polyglutamine repeats in their native context is important for understanding their effects on protein structure and function. In aqueous solution, purified recombinant wt AR NTD (amino acids 1-537) had a relatively limited amount of stable secondary structure. However, for the AR-NTD45Q, a small, but measureable increase in α-helix content and small decrease in the β structures was noted (Davies et al., 2008). The AR-NTD45Q peptide was more sensitive to urea-induced unfolding than the AR-NTD20Q, and limited proteolysis, which was used as a probe for global protein structure, generated a unique pattern of fragments from the polyQ AR NTD. Thus, these spectroscopic and biochemical analyses support the view that the expanded glutamine tract alters the conformation of the AR NTD.

Structural analysis were also performed on full-length AR-proteins expressed using the baculovirus-insect cell system (Jochum et al., 2012). Atomic force microscopy was used to characterize the sub-micrometer scale aggregates of the ARQ22 and ARQ65 proteins purified from DHT-treated Sf9 cells (Jochum et al., 2012). The wt AR formed annular oligomers 120–180 nm in diameter, while the polyQ AR formed oligomeric fibrils up to 300–600 nm in length. Interestingly, ARQ65 purified from DHT- and melatonin-treated Sf9 cells produced annular oligomers, which did not decrease human neuronal SK-N-SH cells viability, unlike the fibrillar oligomer form, suggesting that AR structure modulates pathogenicity.

## Interdomain Interactions

Dynamic interactions between different AR domains are critical for the receptor’s function. DBD-mediated dimerization is essential for the AR to bind DNA and regulate transcription (Centenera et al., 2008). Upon ligand-binding, the AR LBD undergoes a conformational rearrangement that results in formation of a protein interaction surface, the activation function 2 (AF-2) domain, which binds with the ^23^FQNLF^27^ motif near the AR N-terminus. This intermolecular interaction, known as N/C-terminal interaction, is essential for androgen-dependent activation of specific genes (He et al., 2002). To establish whether N/C-terminal interaction of the polyQ AR played a role in aggregation and cell death, the effects of bicalutamide, a transcriptional antagonist of the AR, were tested in a cell model of SBMA and motor neurons from transgenic AR112Q mice (Orr et al., 2010). Bicalutamide not only decreased polyQ AR N/C-terminal interactions, but reduced nuclear inclusion formation in PC12 cells expressing AR112Q, even in the presence of DHT. Furthermore, AR112Q PC12 cells and primary neurons were substantially protected from DHT-induced cell death by the addition of bicalutamide. Mutations in the ^23^FQNLF^27^ motif of AR112Q also reduced DHT-dependent cell toxicity and polyQ AR aggregation, suggesting that N/C-terminal interaction must be maintained in order for the polyQ AR to exhibit its pathogenic gain-of-function properties.

## Altered Protein Interactions

**Figure 2:**
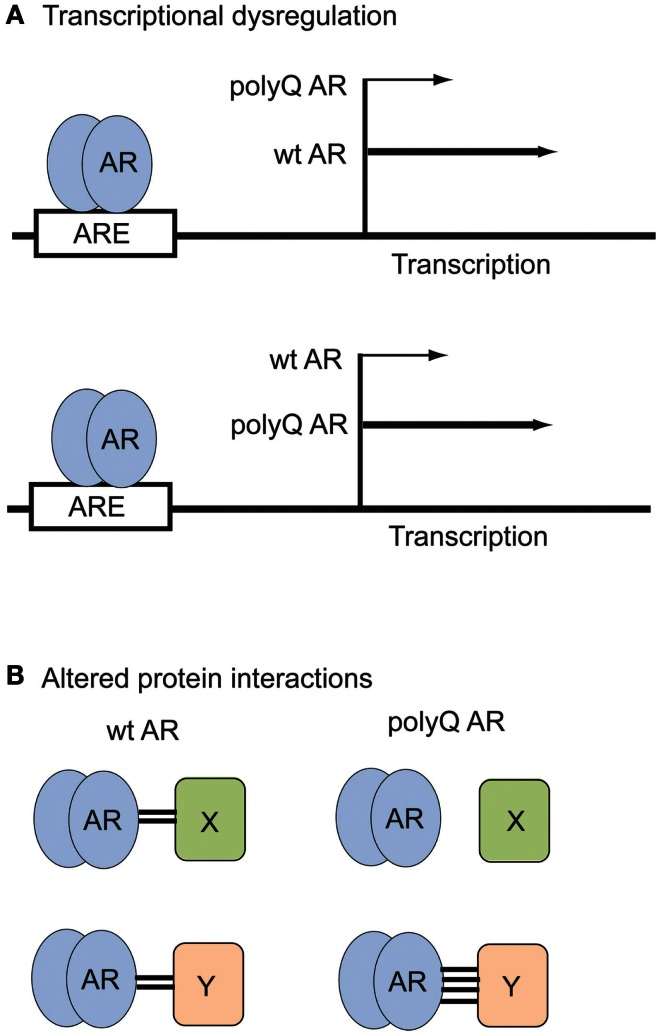
**Examples of polyQ AR loss- and gain-of-function in SBMA**. **(A)** Transcriptional dysregulation. The polyQ AR may activate expression of certain genes to a lesser (top) or greater (bottom) extent than the wt AR. **(B)** Altered protein interactions. The polyQ AR may fail to associate with proteins (top) or bind more strongly to proteins (bottom) that normally interact with the wt AR.

The AR NTD contains a transactivation domain that participates in multiple protein–protein interactions with general transcription factors and co-regulatory proteins. Thus changes in the AR NTD induced by polyglutamine expansion (Davies et al., 2008) can potentially strengthen or diminish the interaction of the polyQ AR with these proteins (Figure 2). Overall, the wt AR is known to interact directly with more than 236 distinct proteins (Gottlieb et al., 2012). For instance, the SIRT1 deacetylase binds to the AR (Fu et al., 2006), and modulates aggregation and polyQ AR proteotoxicity (Montie et al., 2011). Several proteins differentially interact with the wt or polyQ AR, implicating different pathways in SBMA pathogenesis. While cytochrome c oxidase subunit Vb (COXVb) interacted more strongly with wt AR than polyQ AR in a hormone-dependent manner, COXVb co-localized with polyQ AR, but not wt AR aggregates (Beauchemin et al., 2001), which may contribute to mitochondrial dysfunction in SBMA. In the presence of DHT, retinoblastoma protein (Rb) weakly associated with wt AR, but interacted strongly with the polyQ AR, leading to aberrant E2F1 transcriptional activation (Suzuki et al., 2009). The p23 protein, an essential component of the multichaperone Hsp90 complex, was more highly associated with the polyQ AR than the wt AR, which may be the basis for the preferential degradation of the polyQ AR after 17-AAG treatment (Waza et al., 2005). AR113Q, but not AR10Q, specifically interacted with PTIP (Pax Transactivation-domain-interaction Protein), a protein that functions in DNA repair, suggesting the polyQ AR may dampen the DNA damage response, leading to an accumulation of mutation and cellular dysfunction in SBMA (Xiao et al., 2012). The full impact on cellular functions mediated by novel or altered interactions between the polyQ AR and other proteins is still being explored.

## Nuclear Translocation

The ligand-dependent translocation of AR to the nucleus is required for the receptor to carry out its classical functions as a transcription factor. Transgenic mice expressing a polyQ AR with a deletion within the AR bipartite NLS (ARdNLS112Q) exhibited delayed onset and reduction of motor deficits compared to AR112Q mice. The nuclear accumulation of ARdNLS112Q in the spinal motor neurons of the ARdNLS112Q mice was also deferred relative to the AR112Q mice. Interestingly, in PC12 cells expressing a polyQ AR with a constitutive-acting NLS (NLSX3-AR76Q), although the NLSX3-AR76Q protein localized to the nucleus in the absence of DHT, neither intranuclear inclusions nor cell death were observed. However, upon DHT treatment of the NLSX3-AR76Q cells, inclusions consisting of N-terminal AR fragment formed, and levels of cell death increased.

In a *Drosophila* model, transgenic flies that expressed the AR73Q protein with mutations in the NLS (K633A and K634A; AR73Q KK/AA) were generated (Nedelsky et al., 2010). In addition, a polyQ AR incapable of binding DHT (S215D and S792D mutations; AR65Q SS/DD) was fused to a NLS, to produce NLS-AR65Q SS/DD transgenic flies. *Drosophila* expressing AR52Q or AR65Q-NLS developed an SBMA eye phenotype when fed DHT, however, the AR73Q KK/AA or NLS-AR65Q SS/DD flies showed no signs of photoreceptor degeneration, even when raised on DHT. Overall, these results from these two models indicate that the polyQ AR must be localized within the nucleus, in the presence of its ligand, to induce SBMA disease features.

## Aggregates and Nuclear Inclusions

The accumulation of misfolded and aggregated proteins is a pathological hallmark of many neurodegenerative diseases, including ALS and polyglutamine diseases (Pandey et al., 2007a). However, immunohistochemical examination of tissues from SBMA patients with the 1C2 antibody (a monoclonal antibody that specifically recognizes expanded polyglutamine tracts) showed that diffuse nuclear accumulation of the polyQ AR was far more frequent and extensive than nuclear inclusions, was distributed in a wide array of CNS nuclei, and present in more visceral organs than previously identified (Adachi et al., 2005). Cytoplasmic accumulations of the polyQ AR were also detected in neural and non-neural tissues (Adachi et al., 2005). Although there are conflicting views on whether polyQ AR aggregates or nuclear inclusions have a detrimental or a protective effect (Taylor et al., 2003; Beitel et al., 2005), these aggregates may be an indicator of the intracellular inability to remove misfolded proteins (Li et al., 2008). Furthermore, the 1C2-reactive neuronal inclusions can be detected using antibodies directed against specific AR N-terminal regions, but not the DNA- or ligand-binding domains, strongly suggesting that proteolytic cleavage of the polyQ AR generates truncated fragments that are incorporated into the inclusions (Butler et al., 1998; Li et al., 1998; Merry et al., 1998; Chevalier-Larsen et al., 2004). As well, numerous cellular protein have been found to be sequestered into neuronal intranuclear inclusions including HSPs (Hsp40, Hsp70, Hsp90), UPS components (ubiquitin, 19S and 20S proteasome core proteins), and several AR co-activators and transcriptional proteins (CBP, SRC-1) (Rusmini et al., 2011; Schindler et al., 2012).

The first 50 amino acids of the polyQ AR (e.g., filamentous actin-binding sequences) can directly affect aggregation rate, the SDS solubility and the subcellular localization of aggregates (Angeli et al., 2010). Using a FRET assay to measure aggregation of a polypeptide containing the first 127 amino acids of AR, it was determined that coexpression of profilin, an actin-binding protein, inhibited polyQ AR aggregation (Shao et al., 2008). Conversely, cofilin, an F-actin-severing and -depolymerizing factor, increased aggregation, suggesting that the actin cytoskeleton activity may influence polyQ AR aggregation. Disrupting interaction between AR and the ARA70 coactivator in PC12/AR10Q cells with ASC-J9 (5-hydroxy-1,7-bis(3,4-dimethoxyphenyl)-1,4,6-heptatrien-3-one) promoted AR degradation (Yang et al., 2007). ASC-J9 also reduced aggregate formation and polyQ AR-induced cell death in PC12 AR112Q cells, and in AR97Q transgenic mice reversed muscular atrophy, and improved motor impairment and lifespan. Similar effects were seen in neuronal cells and transgenic mice when genistein, a soy isoflavone, was used to disrupt polyQ AR-ARA70 interactions (Qiang et al., 2013). Thus, decreasing aggregate formation by treatment with compounds that disrupt AR-protein interactions may allow the polyQ AR to be degraded, with beneficial effects.

## Formation of Soluble Oligomers

In several SBMA models, the presence of large intracellular inclusions does not correlate with neuronal toxicity or SBMA phenotype (Table 1), suggesting other forms of the polyQ AR may be pathogenic. For example, expression of N-terminal truncated polyQ AR fragments in neuronal cells activated c-jun N-terminal kinase (JNK), leading to phosphorylation of c-Jun, which then initiated a Bax-dependent apoptotic cascade (Young et al., 2009). It has also been proposed that soluble polyQ AR NTD fragments can interact with the full-length polyQ AR and impair its transactivation capacity (Schiffer et al., 2008). Although intracellular polyQ AR aggregates are identified using histopathological methods, oligomers have been defined as submacromolecular structures that are soluble after high-speed centrifugation and are comprised of ordered polyglutamine aggregates (Li et al., 2007). In brain extracts from symptomatic male AR112Q mice, the AR112 monomer (∼130 kDa) and an intermediate molecular weight smear (∼250–450 kDa) suggestive of soluble oligomers of polyQ AR protein, were identified by Western blotting (Li et al., 2007). Formic acid treatment has been shown to dissociate SDS-insoluble protein aggregates stabilized by hydrogen bonds, but does not cleave isopeptide bonds. Treatment with formic acid dissociated the polyQ oligomers from SBMA mice and indicated that the oligomers contained a distinct polyQ AR N-terminal fragment of ∼50 kDa. In the male AR112 mice, the oligomers appeared before the onset of motor dysfunction, and did not correlate with the occurrence of nuclear inclusions. However, upon castration, which reversed polyQ-induced pathology in the male AR112 mice, the levels of oligomers decreased rapidly, further implicating soluble polyQ AR oligomers in SBMA pathogenesis.

## Post-Translational Modifications

AR function, trafficking, and turnover are dynamically regulated by a variety of post-translational modifications including phosphorylation, acetylation, ubiquitination, sumoylation, and methylation (Figure 1) (Montie et al., 2011; Anbalagan et al., 2012; Coffey and Robson, 2012). These modifications can occur through both androgen-independent and androgen-dependent mechanisms. Notably, the polyQ AR was found to be acetylated and phosphorylated in the absence of ligand, while the wt AR underwent these post-translational modifications only in the presence of ligand (Lieberman et al., 2002).

In cells expressing the polyQ AR, phosphorylation of AR112 at S516 via the p44/42 MAP kinase pathway was required to induce cell death (LaFevre-Bernt and Ellerby, 2003). It was proposed that this phosphorylation event enhanced the ability of caspase-3 to cleave the AR112Q and generate cytotoxic polyQ fragments. Akt (also known as protein kinase B) phosphorylates the wt AR at S215 and S792 (Lin et al., 2001). Phosphorylation of AR65Q at the same Akt consensus sites reduced androgen binding and transcriptional activation, and decreased ligand-induced protein stabilization and nuclear translocation (Palazzolo et al., 2007). Insulin-like growth factor 1 (IGF-1) stimulation increased the survival rate of the MN-1 cells stably expressing AR65Q through the phospho-inositol-3-kinase (PI3K) pathway and activation of Akt. IGF-1/Akt signaling also reduced polyQ AR aggregation and increased polyQ AR degradation by the UPS in a phosphorylation-dependent manner (Palazzolo et al., 2009). To study the effects of IGF-1 *in vivo*, transgenic AR97Q mice were crossed with mice overexpressing a rat, non-circulating, muscle-specific isoform of IGF-1. The AR97Q/IGF-1 mice had reduced polyQ AR aggregation in muscles and spinal cord, decreased muscle weakness, less motor neuron loss, improved motor function, and extended life span compared to AR97Q mice, strongly suggesting that augmentation of IGF-1/Akt signaling could counteract the phenotypic and pathological abnormalities caused by the polyQ AR. Further studies showed systemic treatment of symptomatic AR97Q mice with recombinant human IGF-1 and IGF-1 binding protein 3 (rhIGF-IGFBP3) resulted in increased Akt activation, reduced polyQ AR aggregation and pathology in muscles, improved motor function and increased survival (Rinaldi et al., 2012). Phosphorylation at Akt sites can thus modulate the detrimental effects of the polyQ AR.

Acetylation of wt AR at three lysine residues (K631, K633, and K634) within the AR hinge region is regulated by several acetyltransferases including p300, p300/CBP-associated factor (P/CAF), and TIF60 (Tat-interactive protein). Androgen-induced acetylation of AR can regulate DNA-binding and enhances AR transcriptional activity in a promoter-dependent context (Anbalagan et al., 2012; Coffey and Robson, 2012). Conversely, deacetylation downregulates AR activity. SIRT1, a member of the sirtuin family of deacetylases, interacts with and deacetylates the wt AR at the conserved lysine motif, inhibiting DHT-dependent wt AR signaling and N/C interaction (Fu et al., 2006). PC12 cells inducibly expressing AR112Q form polyQ length and DHT-dependent nuclear inclusions of proteolyzed polyQ AR, and die in response to DHT (Montie et al., 2011). In the presence of androgens, nuclear-localized AR112Q was hyperacetylated compared to the AR10Q. Stable overexpression of SIRT1 in this model reduced the number of cells with nuclear inclusions and the amount of SDS-insoluble AR112Q seen on Western analysis. SIRT1 overexpression also protected PC12 cells expressing AR112Q, and motor neurons from AR112Q mice, from DHT-dependent death, demonstrating that abnormal acetylation is associated with aberrant polyQ AR function in SBMA.

Sumoylation, the process of covalently attaching small ubiquitin-like modifier (SUMO) to proteins, is mediated by activating, conjugating and ligating enzymes, and reversed by a family of SUMO-specific proteases (SENPs) (Anbalagan et al., 2012). Sumoylation of the wt AR at K388 and K521 in the NTD can inhibit AR transcriptional activity in a promoter context-dependent manner (Mukherjee et al., 2009). Expansion of the polyglutamine tract in the AR does not appear to interfere with AR sumoylation. However, increasing SUMO levels in AR113Q-expressing HeLa cells decreased the fraction of cells containing ligand-induced AR aggregates, without affecting total polyQ AR levels. Thus, sumoylation reduced the formation of polyQ AR aggregates independently of its effect on AR113Q transcription activity.

## Transcriptional Dysregulation

Dysregulation of transcriptional events is considered to be a major molecular mechanism through which the polyQ AR contributes to the development of SBMA. The pathogenic polyQ AR accumulates in the cell nucleus in a ligand-dependent manner and may inhibit transcription by interfering with the function of essential transcriptional factors and co-activators (Katsuno et al., 2005). For example, in SBMA cell models, transgenic mice and tissues from SBMA patients, transcriptional co-activators such as CREB-binding protein (CBP) can be sequestered into nuclear inclusions formed by the polyQ AR (McCampbell et al., 2000). AR transcriptional competence has been found to decrease as polyglutamine tract length increases (Kazemi-Esfarjani et al., 1995; Thomas et al., 2006b; Palazzolo et al., 2008). Microarray analysis of MN-1 cells stably expressing 24Q or 65Q AR showed that specific genes that were up- or down-regulated in an androgen-responsive fashion by the wt AR failed to be activated by the polyQ AR, indicative of a partial loss of AR transcriptional activity (Lieberman et al., 2002) (Figure 2). In NSC34 cells, the polyQ AR was found to act in DHT-dependent promoter specific context, as ARQ.48 was less effective than AR.Q22 in inducing transcription from classical AREs, but acted similarly to AR.Q22 in repressing transcription from a promoter with a non-classical ARE silencer region (Vismara et al., 2009).

In mouse models of SBMA, decreases in the mRNA levels of vascular endothelial growth factor (VEGF) (Sopher et al., 2004), skeletal muscle chloride channel 1 (CLCN1), skeletal muscle sodium channel α-subunit, neurotrophin-4 (NT-4), glial cell line-derived neurotrophic factor (GDNF) (Yu et al., 2006a), dynactin 1 (Katsuno et al., 2006), genes related to mitochondrial function (Ranganathan et al., 2009), and TGF-β receptor type II (TGF-βRII) (Katsuno et al., 2010a) have been described. Gene expression analysis by qPCR showed muscles from men with SBMA and male AR113Q mice, but not spinal cords or spinal motor neurons, contained significantly higher levels of several mRNAs that are induced in response to endoplasmic reticulum (ER) stress (Yu et al., 2011). Microarray analysis of spinal cords of AR97Q mice showed the gene encoding calcitonin gene-related peptide α (CGRP1) was upregulated, and mediated neuronal damage via activation of the JNK pathway (Minamiyama et al., 2012). Interestingly, although mutations in the FUS (fused in sarcoma) gene are associated with ALS, no evidence of altered FUS expression was found in the spinal cord or motor neurons of AR100 mice (Fratta et al., 2013).

A global characterization of gene expression in three mice models that substantially reproduce an SBMA phenotype was also pursued (Mo et al., 2010). Microarray analyses of the lower hind limb muscles of AR113Q knock-in, AR97Q transgenic, and HSA-AR transgenic mice, relative to wt mice, were carried out. The patterns and levels of transcription of a number of genes were found to be altered in all models. In the polyQ AR models, genes functionally associated with metal ion binding, steroid metabolism, muscle contraction, filamentous actin, endocytosis, chemical homeostasis, and macrophage activation were found to be down-regulated, while genes involved in hydrolase activity, protein binding, and peptidase activity were upregulated. These results support the hypothesis that altered gene expression due to the presence of the polyQ AR may underlie the muscle wasting and motor neuron loss seen in SBMA.

Intriguingly, microRNA (miRNA) expression can also be altered due to the polyQ AR (Miyazaki et al., 2012). Over 500 miRNAs were analyzed using microarrays, and five miRNAs were upregulated more than twofold in the spinal cord of AR97Q mice at an advanced disease stage relative to the AR 24Q mice. At this point, the mechanisms by which the polyQ AR up-regulates miRNAs and the consequences are unknown.

## Altered RNA Splicing

Alternative splicing allows for the synthesis of different products from the same gene, increasing the diversity of proteins that can be generated from a limited number of genes. The role of the wt AR in RNA processing and splicing is presently unclear, although the AR has been shown to interact in a ligand-dependent manner with RNA splicing factors PTD-associated (PSF) and p54nrb (Dong et al., 2007). Hormone- and glutamine length-dependent missplicing of the chloride channel 1 (*Clcn1*) gene and increased expression of the CUGBP1 RNA-binding protein has been demonstrated in SBMA AR113Q knock-in mice (Yu et al., 2009). In AR21Q mice, however, skeletal muscle denervation also induced CUGBP1, but did not alter *Clcn1* RNA splicing, suggesting that a combination of denervation- and polyQ AR-mediated mechanisms is required to alter RNA processing in SBMA model mice (Yu et al., 2009). In *in vitro* studies, the human cardiac troponin T (cTNT) minigene construct was transfected into HeLa cells as a reporter to determine the effect of the wt or polyQ AR on RNA splicing. Expression of the AR24Q in the presence of ligand or the AR112Q in the absence or presence of ligand resulted in increased cTNT exon 5 inclusion. Transcription and RNA processing of the mouse mammary tumor virus (MMTV) driven-calcitonin/calcitonin gene-related peptide (CT/CGRP) minigene is affected by steroid hormone receptors (Auboeuf et al., 2007). Although activation of the AR24Q by ligand slightly increased the CT/CGRP transcript ratios, ligand activation of AR112Q caused a significantly greater increase in this ratio (Yu et al., 2009). Thus, the polyQ AR can influence androgen-regulated RNA processing and splicing events, although the precise effects of altered RNA metabolism on cellular function in SBMA patients remain to be determined.

## Ubiquitin Proteasome System Impairment

Protein homeostasis is regulated in the cell by two principal degradative mechanisms: the ubiquitin proteasome system (UPS), which processes short lived and misfolded proteins for degradation (Ciechanover and Brundin, 2003), and autophagy (Rubinsztein, 2006). Ubiquitination is the enzymatic process that occurs when a ubiquitin moiety is attached to a protein via a highly structured enzymatic cascade that involve an activating enzyme (E1), a conjugating enzyme (E2), and a ubiquitin ligase enzyme (E3) that is substrate specific (Ciechanover and Brundin, 2003). The wt AR-protein undergoes post-translational modifications that modulate its activity, including ubiquitination (Coffey and Robson, 2012; Gioeli and Paschal, 2012) (Figure 1). Mass spectrometry analyses have found two ubiquitination sites in the AR protein, K846, and K848 (Xu et al., 2009). Ubiquitin ligases identified for the AR include MDM2 (Lin et al., 2002), C-terminus of heat-shock cognate protein 70 (Hsc70)-interacting protein (CHIP) (Chymkowitch et al., 2011) and RNF6 (Xu et al., 2009). MDM2 and CHIP target the AR for degradation by polyubiquitinating it, while ubiquitination by RNF6 promotes AR-dependent transcription (Xu et al., 2009). Additional UPS proteins that interact directly with and coactivate the wt AR are an E2 (UbcH7), several E3s (ARA54, E6-AP, hPIRH2/ARNIP), a deubiquitinating enzyme (USP10), and a proteasomal subunit (Rpt5/PSMC3) (Gottlieb et al., 2012). AR function and the UPS may therefore overlap in a number of areas, beyond proteasome-mediated degradation.

One of the characteristic features of the neurodegenerative polyglutamine diseases is the presence of inclusion bodies that colocalize with components of the UPS and the molecular chaperone pathway (Adachi et al., 2001), indicating that the mutant proteins are targeted for degradation (Stenoien et al., 1999). Potentially, sequestration of UPS components or inhibition of the UPS by polyQ proteins could alter protein repair and degradation pathways and thus have been implicated in the pathogenesis of polyglutamine diseases (Bailey et al., 2002; Rusmini et al., 2010). Degradation of an unstable green fluorescent protein reporter (GFP^u^) decreased when HEK293-GFP^u^ cells expressing the polyQ AR were treated with androgens, suggesting that proteasome activity was inhibited by the polyQ AR (Mandrusiak et al., 2003). Androgen-dependent polyQ AR (121Q) UPS impairment was also observed in SBMA flies expressing a reporter for proteasome function (Pandey et al., 2007b). In contrast, in an NSC34 cell model of SBMA, unliganded soluble polyQ impaired the cytoplasmic UPS, and, although testosterone binding induced polyQ AR aggregation, nuclear UPS activity was unaffected (Rusmini et al., 2007). Proteasomal proteolytic activity was also preserved in AR97Q mice, even when they developed a severe phenotype (Tokui et al., 2009). At this point, the complex processes that may lead to proteasomal inhibition and the extent to which UPS dysfunction contribute to SBMA require further investigation.

## Autophagy

Macroautophagy, often simply called autophagy, is the process by which misfolded or damaged proteins are targeted for degradation through the lysosomal system (Rubinsztein, 2006). The role of autophagy has been assessed in several SBMA models. Transgenic *Drosophila* expressing full-length hAR with 52Q or 121Q displayed ligand-dependent, polyQ AR-dependent degeneration of specific neurons, resulting in a rough eye phenotype (Pandey et al., 2007b). This phenotype was associated with UPS impairment and induction of morphological features of autophagy. Inducing autophagy in this fly model, through overexpression of histone deacetylase 6 (HDAC6), accelerated the turnover of the polyQ AR and lowered steady-state levels of monomeric and aggregated polyQ AR. Treatment with the TOR inhibitor rapamycin also suppressed degeneration, suggesting a compensatory relationship between autophagy and the UPS. Activation of autophagy in motor neurons from AR112Q mice also prevented DHT-dependent death induced by nuclear-localized polyQ AR, although, unlike in the fly model, a reduction in the levels of monomeric polyQ AR was not seen (Montie et al., 2009). In NSC34 cells, 17-AAG (17-allylamino, 17-demethoxygeldamycin) treatment promoted the solubility and degradation of the misfolded AR.Q46 through the autophagic pathway, without affecting the UPS (Rusmini et al., 2011).

The unfolded protein response (UPR), an ER protein quality control pathway, was found to be induced in skeletal muscle from SBMA patients and male AR113Q knock-in mice (Yu et al., 2011). Unexpectedly, genetic disruption of the UPR in the AR113Q mice worsened skeletal muscle atrophy, even though autophagy was activated. Conversely, impairing autophagy decreased muscle wasting and prolonged survival of the SBMA mice. The contrasting results seen with the pharmacological and genetic manipulation of autophagy in these SBMA models highlights the complexity of the relationship between the UPR, autophagy, and the UPS, as well as the ability of these systems to clear the polyQ AR from different tissues and subcellular compartments.

## Heat-Shock Proteins

Heat-shock proteins are molecular chaperones that facilitate the refolding, assembly and intercellular transport of proteins. The wt AR is normally stabilized by the chaperone activity of Hsp90/Hsp70 complex, which maintains the AR in a conformation that permits androgen binding (Wang et al., 2010). Overexpression of Hsp70, and Hsp40, inhibited accumulation of the polyQ AR protein and suppress cell death in several cells models (discussed in Beitel et al., 2005; Katsuno et al., 2005). Oral treatment of transgenic AR97Q mice with geranylgeranylacetone (GGA), which induced Hsp70, Hsp90, and Hsp105 expression in the central nervous system and skeletal muscle, inhibited the nuclear accumulation of the polyQ AR, and ameliorated the neuromuscular phenotype (Katsuno et al., 2005). Treatment of AR100 mice with arimoclomol, a co-inducer of the heat-shock stress response, upregulated Hsp70 in spinal cord and hindlimb muscle, improved neuromuscular function and motor neuron survival, and delayed disease progression (Malik et al., 2013). Overexpression of Hsp70 interacting protein (Hip), a co-chaperone that stabilizes Hsp70, in AR112Q-expressing HeLa cells enhanced clearance of soluble and ligand-induced insoluble polyQ AR proteins through the UPS (Wang et al., 2013). Similarly, a small molecule (YM-1) that converted Hsp70 to its tight-affinity conformation, increased Hsp70-dependent degradation of hormone-induced AR113Q oligomers in tetracycline-inducible PC12 cells, and partially rescued the DHT-dependent rough eye phenotype in UAS-AR52 flies (Wang et al., 2013). In contrast, inhibition of Hsp70 by methylene blue impaired degradation and enhanced aggregation of full-length AR112Q in HeLa cells (Wang et al., 2010).

The Hsp90/Hsp70-based chaperone machinery is part of the cellular defense against unfolded proteins (Morishima et al., 2008). When Hsp90 heterocomplex assembly is blocked by specific Hsp90 inhibitors like geldanamycin, the client proteins undergo rapid degradation through UPS, assisted by E3 ligases such as CHIP. Indeed, CHIP functions as a negative regulator of wt AR transcriptional activity by promoting AR degradation (He et al., 2004). In SH-SY5Y cells, increasing levels of CHIP effectively ubiquitinated and degraded the monomeric polyQ AR more efficiently than the wt AR, suggesting that the polyQ AR is more sensitive to CHIP than the wt AR (Adachi et al., 2007). Overexpression of CHIP in AR97Q transgenic mice also inhibited neuronal nuclear accumulation of polyQ AR and ameliorated motor symptoms (Adachi et al., 2007). However, the AR112Q protein was degraded at the same rate in CHIP-deficient and CHIP-positive mouse embryonic fibroblasts after treatment with geldanamycin, strongly suggesting that redundant E3 ligases promote polyQ AR degradation through the UPS (Morishima et al., 2008). Further analysis indicated that Hsp90 inhibition by geldanamycin in heat-shock transcription factor (*Hsf1)*-null mouse embryonic fibroblasts did not increase Hsp70 and Hsp40 levels, but still prevented AR112Q aggregation, possibly by inhibiting Hsp90-mediated retrograde trafficking (Thomas et al., 2006a).

A less toxic derivative of geldanamycin, 17-AAG, also shifts the Hsp90 complex toward its proteasome-targeting form, leading to enhanced UPS degradation of Hsp90 client proteins. 17-AAG treatment preferentially degraded the polyQ AR relative to the wt AR in both SH-SY5Y cells, and spinal cord and muscle of transgenic AR97Q mice, thus reducing monomeric and aggregated polyQ AR levels, and decreasing motor impairment in AR97Q mice (Waza et al., 2005). Similar effects were seen in cells and AR97Q mice treated with 17-(dimethylaminoetylamino)-17-demthoxygeldanamycin (17-DMAG), a more potent derivative of 17-AAG (Tokui et al., 2009). Manipulation of Hsp70 and Hsp90 activity can therefore influence poly Q AR stability and degradation.

## Loss of Neurotrophic Support

Initially, it was postulated that the polyQ AR gained functions that were harmful to motor neurons, resulting in neuronal loss and denervation-induced muscular atrophy in SBMA. However, another mechanism contributing to neuronal degeneration observed might be the loss of AR-dependent functions that support neuronal cell survival. Although each polyglutamine disease appears to specifically affect different subsets of neurons, SBMA is the only one to cause selective degeneration of motor neurons (Orr and Zoghbi, 2007). As described above, AR100Q mice developed a more severe motor neuron phenotype in the absence of the endogenous wt AR (Thomas et al., 2006b). This implies that the wt AR may play a role in supporting motor neurons and that the polyQ AR is dysfunctional with respect to protecting the neurons from degeneration (Cary and La Spada, 2008). Neurotrophins, including brain-derived neurotrophic factor (BDNF), ciliary neurotrophic factor (CNTF), and GDNF, are small peptide hormones that promote motor neuronal growth and survival (Cary and La Spada, 2008). VEGF and IGF-1 also display motor neuron-specific trophism. Muscles from SBMA patients showed decreased expression of GDNF (Yamamoto et al., 1999). Decreased expression of GDNF, IGF-1, and NT-4 were seen in the AR113Q knock-in mice (Yu et al., 2006a), while lower VEGF levels were found in the AR 100Q YAC mouse model (Sopher et al., 2004). Signaling through the AR itself could also protect specific motor neurons by inducing transcription of genes involved in promoting motor neuron survival (Cary and La Spada, 2008). It is also thought that the BDNF produced by muscle can be transported in a retrograde fashion and thus act as a trophic factor to support motor neurons (Fargo et al., 2008).

The lower motor neurons in the spinal cord and brainstem express high levels of AR, and growth of their elaborate dendritic arbors and extremely long axons is developmentally regulated by androgens (Fargo et al., 2008). In NSC34 cells stably transfected with mouse wt AR, androgens enhanced motor neuron differentiation and neurite outgrowth, likely through upregulation of neuritin (Marron et al., 2005). Motor neurons expressing the polyQ AR, however, developed neurites that were typically short and dystrophic, with abnormal branching patterns (Poletti, 1999). These results suggest that the polyQ AR may prevent the normal pattern of connectivity of motor neurons by interfering with control of their neurite outgrowth. The loss of connectivity with the target musculature could eventually result in death of the motor neurons (Fargo et al., 2008). As well, the polyQ AR may fail to upregulate neuritin, which otherwise might mitigate the detrimental effects of polyQ AR aggregates and allow the motor neurons to maintain or recover normal axonal functionality (Fargo et al., 2008).

## Myogenic Mechanisms

The supposition that SBMA is of neurogenic origin (i.e., originates from neuronal degeneration) has been broadened to include the possibility that this disorder may also have a myogenic contribution. The AR113Q knock-in mice developed early androgen-dependent neuromuscular weakness, and myopathic and neurogenic skeletal muscle pathology was observed before neuronal intranuclear inclusions were seen in the spinal cord (Yu et al., 2006a). Unexpectedly, transgenic mice overexpressing the wt AR in skeletal muscle, but not spinal motor neurons (HSA-AR; Table 1), developed an androgen-dependent SBMA-like phenotype (Monks et al., 2007). Although prominent nuclear staining for AR in muscle fibers was observed, there was no evidence for AR-positive aggregates. The male HSA-AR mice exhibited a phenotype similar to other SBMA mouse models, with kyphosis, reduced body weight, weakness, and motor dysfunction. The skeletal muscles of the HSA-AR mice show pathological abnormalities consistent with an SBMA phenotype (myopathy), and signs of motor axon loss, but no loss in the number of motor neuron cell bodies. Myogenin and acetylcholine receptor (AChR) α-subunit mRNA levels were upregulated in HSA-AR muscles, consistent with neurogenic atrophy, while VEGF, a candidate trophic factor for motor neurons, was down-regulated. These results raised the prospect that diseases such as SBMA that have been regarded as “motor neuron diseases” result from processes that originate in muscle, and eventually cause pathology in motor neurons, possibly due to loss of muscle-derived neurotrophic factors (Monks et al., 2007, 2008; Yu et al., 2011).

HSA-AR female transgenic mice were asymptomatic, but rapidly lost motor function when exposed to male levels of testosterone (Johansen et al., 2009). Neither motor neuron nor muscle fiber losses were seen, however, motor deficits were associated with androgen-dependent changes in muscle gene expression. Furthermore, the HSA-AR mice were crossed with *tfm* mice to generate tfm/HSA-AR mice with functional AR only in skeletal muscles (Johansen et al., 2011). The tfm/HSA-AR males had *tfm*-like external genitalia, undescended atrophic testis, low levels of circulating testosterone, but no signs of an SBMA phenotype. Upon testosterone treatment, however, these mice developed a profound neuromuscular phenotype, even though they lacked AR expression in motor neurons. When testosterone treatment was stopped after 9 days, the tfm/HSA-AR males rapidly regained motor function and body weight. Overall, these experiments suggest that cellular dysfunction, rather than loss, underlie the motor deficits triggered by testosterone in the HSA-AR mice.

Investigating the AR113Q mice has also shed light on the role of the polyQ AR in the neuromuscular weakness seen in SBMA (Yu et al., 2011). The male AR113Q mice had less forelimb strength compared to their wt littermates, and morphological changes in skeletal muscle were indicative of both neurogenic and myopathic effects. As in the HSA-AR mice, the skeletal muscles of the AR113Q expressed higher levels of myogenin and AChR α-subunit compared with wt muscle, reflective of denervation. Intranuclear inclusions were detected in muscles of AR113Q males before neuronal intranuclear inclusions developed in their spinal cord. These results suggest that SBMA is initiated by myopathic effects in skeletal muscle, combined with either functional denervation or distal axonopathy that is reflective of motor neuron dysfunction, and that motor neuron loss is a late manifestation of SBMA (Yu et al., 2011).

## Non-Genomic AR Signaling

Androgens can stimulate cell growth and survival through both genomic and non-genomic pathways (Foradori et al., 2008). In the classical view, most androgen action is mediated by an intracellular AR acting at the genomic level as a transcription factor. However, the wt AR is also known to interact with second messenger signaling cascades, such as the mitogen-activated protein kinase (MAPK) pathway (Cary and La Spada, 2008). Investigations into signaling pathways modulated by membrane-associated ARs were performed by inducing expression of AR20Q and AR51Q in NSC34 cells (Schindler et al., 2012). The NSC34/AR51Q cells formed neither insoluble cytoplasmic aggregates nor nuclear inclusions, however, both neurite outgrowth and cell viability were decreased compared to AR20Q-expressing cells. Both the wt AR and polyQ AR localized to the plasma membrane and migrated to lipid rafts upon testosterone treatment, but only the AR20Q activated c-jun, through the JNK pathway. Activated c-jun is involved in neurite outgrowth and cell proliferation, thus these results suggest that the impairment of non-genomic AR signaling may be involved in the development of SBMA.

In addition, an association between polyQ AR-mediated motor neuron damage and disruption of transforming growth factor-β (TGF-β) signaling due to transcriptional dysregulation of TGF-β receptor II (TβRII) has been described. The effects of TGF-β are mediated by transmembrane receptor serine/threonine kinase complex consisting of TβRI and TβRII. TGF-β binding to the complex results in phosphorylation of Smad 2 and 3 (pSmad2/3), which then translocates to the nucleus to regulate gene transcription. Nuclear translocation of pSmad2/3 was suppressed in the spinal cord motor neurons of AR97Q transgenic mice and SBMA patients (Katsuno et al., 2010a). Spinal motor neuron staining and immunoblotting also showed decreased levels of TβRII, but not TβRI, in the SBMA mice and patients. In transfected cells, two factors that regulate TβRII transcription, P/CAF and NF-Y, co-localized with truncated AR97Q in inclusion bodies, potentially causing TβRII down-regulation. As the TGF-β-Smad2/3 signaling pathway has been shown to have potent neuroprotective effects, these results suggest that polyQ-AR-mediated inhibition of TGF-β signaling would result in less effective neuroprotection.

## Mitochondrial Dysfunction

The AR is likely to influence mitochondrial function by regulating transcription of either mitochondrial proteins (encoded by either nuclear or mitochondrial DNA) or transcription factors activating expression of these mitochondrial proteins, or by interacting with proteins that affect mitochondrial function (Gavrilova-Jordan and Price, 2007). Direct AR-mitochondrial interactions are also possible. The wt AR has been localized to mitochondria in human sperm and LNCaP cells (Solakidi et al., 2005), and both the wt AR and, to a greater extent, the polyQ AR associated with mitochondria in cultured MN-1 cells (Ranganathan et al., 2009). The presence of the polyQ AR can alter mitochondrial distribution; cytoplasmic ARQ48 aggregates in transfected HeLa cells sequestered mitochondria, HSPs, proteasome components and steroid receptor coactivator 1 (SRC-1) (Stenoien et al., 1999), and mitochondria were found in polyQ AR aggregates in the NSC34 motor neuron cell line (Piccioni et al., 2002). Both the wt and polyQ AR interacted with COXVb, a nuclear-encoded mitochondrial enzyme involved in oxidative phosphorylation using a mammalian two-hybrid system (Beauchemin et al., 2001). As well, COXVb co-localized to aggregates formed by the polyQ AR in androgen-treated cells, supporting the proposal that sequestration of mitochondrial proteins may lead to mitochondrial dysfunction in SBMA (Beauchemin et al., 2001).

Proliferator-activated receptor gamma coactivator 1 (PCG-1) is known to regulate mitochondrial biogenesis and function. In ligand-treated AR65Q MN-1 cells, PGC-1β mRNA levels were decreased relative to cells expressing the wt AR (Ranganathan et al., 2009). In addition, mitochondrial transcription factor A, a nuclear-encoded gene controlled by PGC-1β, the antioxidant genes superoxide dismutase 1 (SOD1), SOD2, and catalase, and the mitochondrial protein NADH dehydrogenase 1 gene were down-regulated. PGC-1β and SOD2 mRNA levels were also significantly reduced in male AR113Q knock-in mice. Ligand-dependent increases in mitochondrial membrane depolarization, elevated reactive oxygen species (ROS) levels, activation of the mitochondrial caspase pathway, and increased cell death were observed in cells expressing the polyQ AR (Ranganathan et al., 2009). Thus the polyQ AR likely contributes to mitochondrial dysfunction in SBMA directly though abnormal associations with mitochondria or mitochondrial proteins, or indirectly through ligand-dependent alterations in mitochondrial gene expression, turnover, and respiratory function (Beauchemin et al., 2001; Ranganathan et al., 2009). In addition, it has been proposed that mitochondrial DNA damage may be involved in pathogenesis of SBMA or serve as a useful biomarker to monitor disease progression (Su et al., 2010).

## Impaired Axonal Transport

Axonal transport moves proteins and organelles between the neuronal cell body and the axon tip (and vice versa), and is essential for the growth and survival of neurons. Abnormalities in axonal transport have been implicated in neurodegenerative diseases (Morfini et al., 2009; Sau et al., 2011; Ikenaka et al., 2012). Early studies in *in vitro* and cell models suggested that the polyQ AR could hinder axonal transport (Piccioni et al., 2002; Szebenyi et al., 2003). It was shown in SH-SY5Y cells and squid axoplasm that the polyQ AR inhibited fast axonal transport through a pathway that involved JNK activation, phosphorylation of kinesin-1 heavy chain subunits by JNK, and inhibition of kinesin-1 function (Morfini et al., 2006). Transgenic AR97Q mice were found to have impaired retrograde axonal transport, even before the onset of muscle weakness, with accumulation of neurofilaments and synaptophysin in the distal motor axon (Katsuno et al., 2006). In addition, expression levels of dynatin 1, an axon motor for retrograde transport, were reduced in these mice.

In another study, deficits in retrograde labeling of spinal motor neurons were seen in both the AR113Q knock-in and HSA-AR myogenic mouse models of SBMA (Kemp et al., 2011). Live imaging of endosomal trafficking in sciatic nerve axons also showed disease-induced defects in the flux and run length of endosomes movement toward the cell body. However, when axonal transport rates were analyzed in cultured primary motor neurons or in sciatic nerves of AR100 mice, no significant axonal transport deficits were observed, implying there was no correlation between impairment of axonal transport and SBMA pathogenesis (Malik et al., 2011). Further investigation into the discrepancies between different models with respect to polyQ AR-mediated effects on axonal transport is warranted.

## Insights from *Drosophila* Models

The use of genetic models such as *Drosophila melanogaster* has greatly facilitated the study of the underlying molecular mechanisms of SBMA. The development of “humanized” AR transgenic *Drosophila* lines has allowed for the ectopic/tissue-specific expression of the polyQ AR, leading to observable, potentially disease-related phenotypes. Specifically, the wt and polyQ AR genes are cloned into *UAS* expression constructs. Crosses to tissue-specific *GAL4* transgenic fly lines drives the expression of AR in those selected tissues. Although the phenotypes described using such genetic systems are not directly linked to the clinical manifestations of SBMA, the observed phenomena are polyQ tract length-dependent and DHT sensitive. A number of studies have confirmed the working hypothesis of androgen-dependent cellular toxicity of the polyQ AR and have noted the presence of protein aggregates in photoreceptor neurons (Chan et al., 2002; Takeyama et al., 2002; Funderburk et al., 2009; Palazzolo et al., 2010). Interestingly, DHT-dependent reductions in locomotion in larvae expressing the polyQ AR have been described (Funderburk et al., 2009; Nedelsky et al., 2010; Jochum et al., 2012). The role of autophagy and the UPS in a *Drosophila* model of SBMA have also been investigated (Pandey et al., 2007a,b). However, these studies did not address the molecular mechanisms contributing to the observable phenotype or effects on other polyQ AR functions, specifically gene transactivation.

This *Drosophila* genetic system, however, allows for a high-throughput screen of large number of gene sets, and was utilized to screen for genetic interactors that either suppressed or enhanced the polyQ AR/androgen-dependent phenotype (Murata et al., 2008; Suzuki et al., 2009; Nedelsky et al., 2010). Murata et al. (2008) used mutant enhancer trap lines to screen ∼2,000 genes that when co-driven with the polyQ AR would modulate a rough eye phenotype. An RNA-binding protein, *hoip*, was identified that enhanced the polyQ AR-induced eye phenotype (Murata et al., 2008). The homologous gene in yeast has been reported to be involved in rRNA processing (Reichow et al., 2007). By further dissecting the *hoip* protein complex, two more genetic modulators of the polyQ AR phenotype, *nop5* and *nop56* were found. As *nop5* and *nop56* are part of the small nucleolar ribonucleoprotein complex, these results suggest translational regulation may play a role in the neurodegeneration observed in SBMA. Retinoblastoma family protein (*Rbf*), the *Drosophila* homolog of human retinoblastoma protein (Rb), was found to be a neuroprotective factor (Suzuki et al., 2009). Rb is known to function through repressing transcription of genes regulated by the E2F proteins. The androgen-bound polyQ AR, but not the wt AR, appeared to impair transrepressive function of Rb, resulting in aberrant stimulation of E2F-mediated transactivation.

Specific RNAi lines for candidate genes selected from known AR coregulators described in the AR mutations database [http://androgendb.mcgill.ca/; (Gottlieb et al., 2012)] were crossed with AR52Q flies to screen for modifiers of the fly SBMA phenotype. Using this approach, 19 genetic interactors were identified (Nedelsky et al., 2010). Furthermore, microarray analysis was performed on these SBMA flies to identify putative target genes or androgen-regulated genes to account for the observed rough eye phenotype. While a number of differentially expressed genes were observed, neither corresponding AREs nor presumed ecdysone response elements (the sole *Drosophila* steroid hormone receptor) could be identified. Although an interaction of the polyQ AR with a number of genes was proposed, there are a number of post-transcriptional and translation regulatory mechanisms that can alter gene expression profiles. Nevertheless, the approaches taken to carry out high-throughput screens in *Drosophila* are resulting in novel observations of polyQ AR functions that may contribute to SBMA.

## Therapy for SBMA

Given that SBMA is a ligand-dependent polyglutamine disease, clinical trials to reduce testosterone or DHT levels in men have been attempted, but showed only limited improvements in certain disease features (Ranganathan and Fischbeck, 2010; Banno et al., 2012). Nonetheless, investigations into the mechanisms underlying the pathogenesis of SBMA have provided indications for other potential therapies. These include: (1) reducing poly Q AR levels by increasing degradation through Hsp-mediated, UPS or autophagic pathways; (2) decreasing formation of aggregates and/or soluble oligomers, or altering their structure (Jochum et al., 2012); (3) normalizing transcriptional dysfunction; (4) altering abnormal polyQ AR post-translational modifications; (5) decreasing polyQ AR mRNA expression using siRNA or miRNAs (Miyazaki et al., 2012); (6) regulating polyQ AR N/C interactions and nuclear localization; (7) modulating AR-coactivator interactions; (8) using antioxidants to reduce ROS levels; (9) inducing IGF-1/Akt signaling; and (10) treatment with serotonin receptor agonists (Minamiyama et al., 2012). Due to the variety of molecular mechanisms involved in the development of SBMA, a combination of therapies may be needed to reduce symptoms and slow disease progression in men. More details on therapeutic approaches to SBMA may be found in several comprehensive reviews (Ranganathan and Fischbeck, 2010; Banno et al., 2012; Fischbeck, 2012; Katsuno et al., 2012; Tanaka et al., 2012; Rocchi and Pennuto, 2013).

## Conclusion

It is known that androgens can be neurotrophic and are important in muscle development. In retrospect, it is perhaps not surprising that there are both neurodegenerative and neuromuscular components in SBMA, which is caused by polyglutamine tract expansion in the receptor that mediates the actions of androgens. The increase in the length of the polyglutamine tract in the AR is somewhat analogous to the insertion of a new domain through alternative splicing, which generates a novel protein. The polyQ AR retains many of the function of the wt AR, however, it fails to perform as well as the wt AR in certain roles, and gains novel, often deleterious, properties (Figure 2; Table 2). Consequently, polyQ AR-mediated loss- and gain-of-function mechanisms can disturb cellular homeostasis, leading to neuronal and muscular dysfunction. Multiple complex and overlapping pathogenic processes have been shown to contribute to the initiation and development of SBMA. The relative contribution of each mechanistic pathway, and thus the potential for therapeutic intervention, will be the subject for future investigations in this field.

**Table 2:** **PolyQ AR loss- and gain-of-function mechanisms in SBMA**.

Mechanism contributing to SBMA	PolyQ AR *versus* wt AR	
	Loss-of-function	Gain-of-function
Alterations in AR structure		✓
Altered protein interactions	✓	✓
Aggregation		✓
Formation of soluble oligomers		✓
Change in post-translational modifications	✓	✓
Transcriptional dysregulation	✓	✓
Altered RNA splicing	?	?
Ubiquitin proteasome system impairment		✓
Induction of autophagy		✓
Loss of neurotrophic support	✓	
Myogenic contributions	✓	✓
Non-genomic AR signaling	✓	✓
Mitochondrial dysfunction	✓	✓
Impaired axonal transport		✓

## Conflict of Interest Statement

The authors declare that the research was conducted in the absence of any commercial or financial relationships that could be construed as a potential conflict of interest.
